# Basic concepts in facial and neck thread lifting procedures

**DOI:** 10.1111/srt.13673

**Published:** 2024-04-08

**Authors:** Gi‐Woong Hong, Soo‐Bin Kim, Soo Yeon Park, Jovian Wan, Kyu‐Ho Yi

**Affiliations:** ^1^ Samskin Plastic Surgery Clinic Seoul South Korea; ^2^ Division in Anatomy and Developmental Biology Department of Oral Biology Human Identification Research Institute BK21 FOUR Project Yonsei University College of Dentistry Seoul South Korea; ^3^ Made‐Young Plastic Surgery Clinic Seoul South Korea; ^4^ Asia Pacific Aesthetic Academy Hong Kong Hong Kong; ^5^ Maylin Clinic (Apgujeong) Seoul South Korea

**Keywords:** anchoring strength, double chin lifting, facial tissue sagging, facial lifting, holding strength, lateral facial lifting, thread lifting procedures

## Abstract

In this review article, our objective is to elucidate fundamental principles and offer practical illustrations concerning the procedures involved in facial and neck thread lifting. Moreover, we aim to explore associated concepts such as the fixing point, hanging point, and anchoring point terminologies, along with the elucidation of vectors. Additionally, we will provide anatomically oriented explanations of the lifting process required for each facial region using thread lifting methods like V, U, and I techniques using floating type threads (Secrete line, Hyundai Meditech., Inc., Wonjusi, Republic of Korea). Furthermore, our intention is to delve deeply into the concepts of tensile strength, anchoring strength, and holding strength, contextualizing their practical applications within this specific field.

## 1 INTRODUCTION

In the case of thread lifting, addressing the lateral face, which is not significantly correlated with facial expressions in a fundamental sense, could adequately be managed using conventional concepts from existing literature.^1^ However, ensuring comfort related to facial expressions became imperative, necessitating procedures that do not cause discomfort. Particularly for individuals of Asian descent who have prominent cheekbones, the difficulty in accessing the angle between the lateral and anterior faces posed a consistent challenge during procedures, prompting contemplation of various methods to resolve these shortcomings. In recent years, the improvement in the quality of threads manufactured by thread lifting companies, along with the introduction of diverse designs, has contributed to making thread lifting procedures more efficient and yielding superior outcomes compared to the past.

In terms of the anatomical aspects of thread lifting, previously, understanding the relationship between the actual position of threads during procedures and the surrounding structures necessitated cadaver studies and dissections. However, nowadays, the utilization of ultrasound has facilitated a more convenient means of verifying procedural outcomes. Consequently, after attempting a more diverse array of procedures during treatments, it has become possible to assess the results to some extent without directly resorting to cadaver dissections. This development has significantly influenced the enhancement of procedural methods by enabling a more objective evaluation of outcomes, without the need to directly examine cadaveric specimens.^2^, ^3^

The primary objective of thread lifting is often associated with addressing skin and connective tissue sagging that occurs with the progression of aging. Traditionally, there has been a tendency to approach facial aging as a universal sagging of any facial structure following the aging process.^4^ However, the authors argue that fibrous tissues commonly referred to as retaining ligaments in the face do not uniformly succumb to aging but instead maintain their form and strength, contributing to differential effects on various facial regions.^5^

Hence, it is crucial to consider the varying strength of these retaining ligaments, as this disparity can lead to differences in skin and tissue sagging across facial areas.^5^ Understanding these differences is essential to efficiently enhance skin and tissue sagging. It involves determining the appropriate plane for inserting threads, utilizing resilient tissues, and identifying lax tissues to address facial aging mechanisms effectively.

In discussing procedural techniques, authors aim to explore mechanisms that are challenging to resolve through thread lifting, such as sagging tissues. The review will elucidate how specific types of threads can be used to address these issues and describe approaches to improving different facial regions, considering anatomical considerations alongside practical concepts.

Rather than focusing on specific techniques commonly used or individual instructions for various brands of threads in general thread lifting procedures, the review emphasis is on exploring the general mechanisms of action concerning how threads impact tissues during thread lifting.

### 1.1 Definition of the terms

In the context of performing thread‐lifting procedures, it is important to initially address the terminology associated with the procedure. When explaining the types of threads utilized, the procedural techniques, and the mechanisms involved in thread lifting, a range of terminologies is employed beyond medical terms relating to anatomical structures. In this regard, authors aim to elucidate the significance of the terminologies used in our practice, examining whether these terms hold specific meanings or if there are instances of misapplication within the field.

The primary reason notwithstanding, our skin and connective tissues undergo a loss of elasticity and supportive strength with aging, resulting in deepening wrinkles and tissue sagging in the direction of gravity. The primary objective of thread lifting can be described as utilizing threads extensively to pull and secure these sagging tissues in the opposite direction of gravity, preventing their re‐sagging.^6^

The commonly used straight or moderately long floating‐type threads, also known as floating threads, play a pivotal role in this task.^7^ Their protrusions grip onto the lax tissues and are responsible for pulling these tissues in the opposite direction from where they are hanging. Additionally, these threads need to be skillfully placed on the opposite side of the lax tissues to prevent them from falling due to tissue loads. The action of threads' protrusions gripping onto the tissues is referred to as “anchoring.” Through this anchoring action, the loose tissues below are captured by the protrusions of the threads, termed as the “hanging point.” Conversely, areas where the protrusions of the threads are caught in firm tissues are commonly referred to as the “fixing point.”^8^

Therefore, in thread lifting using these protrusion threads, the most critical aspect is the strength of their ability to firmly grasp onto tissues, known as “anchoring strength.” Once the tissues are firmly gripped by the protrusions, the threads need to withstand the load applied by the tissues and external forces to maintain their anchored position. The force applied by the tissues to revert to their original state while being held by the threads' protrusions is termed “stress.” Overcoming this stress, along with the threads' ability to endure and maintain their position, is referred to as “holding.” To ensure long‐lasting procedural effects, the holding strength of these protrusions, signifying the force they withstand to maintain their position, also needs to be substantial.

Previously, there was a tendency to equate anchoring strength and holding strength, assuming that if the protrusions adhered well to tissues, they would maintain their position effectively. However, in the current landscape, where thread manufacturing methods have diversified, with variations in protrusion shapes, positions, directions, and quantities, it is imperative to differentiate and consider both forces separately (Table 1).^10^, ^11^, ^12^, ^13^

**TABLE 1:** The mechanical properties of threads encompass various attributes, notably the definitions of tensile, anchoring, and holding strength.

	Definition
Tensile strength	Tensile strength is gauged by the duration a thread remains intact when subjected to tension force, indicating the force that both holds and pulls the ends of the thread on either side.
Anchoring strength	Anchoring strength signifies the force at which the cog of a thread firmly attaches to the tissue, facilitating the function of pulling and gathering tissue. It involves the cog effectively catching and securing the tissue in place.
Holding strength	Holding strength refers to the capability to sustain forces once the cogs are securely embedded in the tissue, enabling the exertion of force to pull and gather tissue without release.

### 1.2 Lateral facial lifting (short or medium‐length I‐type bidirectional cogged threads)

**FIGURE 1:**
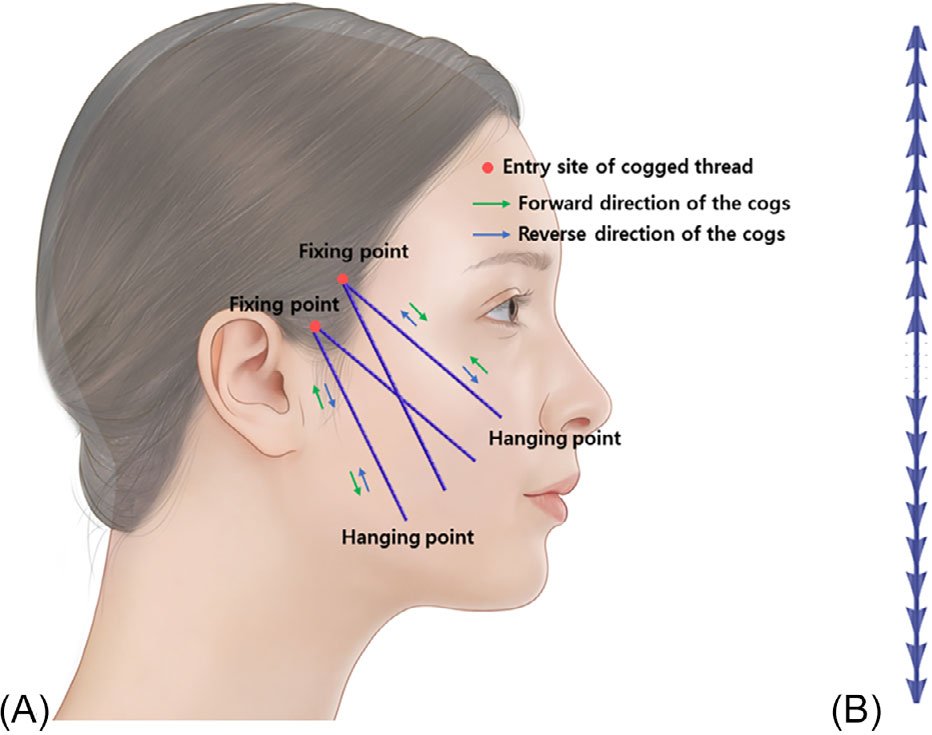
Design of short or medium‐length I‐type bidirectional cogged threads (A). The thread depicted in the image is the Secrete line Illusion (B, Hyundai Meditech., Inc., Wonjusi, Republic of Korea).

In Figure 1, the area where the cogged protrusions of the I‐shaped bidirectional thread attach mainly to the lax tissues below is designated as the “hanging point,” while the region where the thread's protrusions catch onto the firmer tissues above is termed the “fixing point.” Though some refer to this phenomenon as “adhesion” instead of “fixing,” due to the sensation that the threads adhere firmly to tissues, the term “fixing point” is commonly used among medical practitioners to prevent confusion. Irrespective of whether the direction of the protrusions is bidirectional or multidirectional, when using any type of thread, the thread pulling and securing the lax tissues upward should remain fixed and firmly adhered to the upper firm tissues without moving downward. The essential force required here is the “anchoring and holding strength” of the upper protrusions, which need to endure and maintain their position within the firmer tissues, signifying the location where the threads are anchored. To achieve authentic fixation, it would necessitate either securing the threads tightly to the tissues at the base using pins or other means. Consequently, the term “fixing point” we use does not solely imply the degree of attachment or force exerted by the protrusions attaching to the lax tissues below; instead, it predominantly signifies the firm adherence of the upper tissue‐bound protrusions, capable of withstanding significantly greater force. Thus, when inserting straight cogged threads through one upper puncture site, then intertwining and burying the threads emerging from the entry site rather than simply cutting them, it does not strictly equate to securing the threads in place. By interconnecting the protrusions on both sides using straight cogged threads or double‐needle multidirectional threads, it strengthens the fixing point, allowing the threads on the sides to assist each other in better withstanding the downward force caused by the lax tissues held by the protrusions, enhancing the fixing point. This technique, often referred to as “suspension,” highlights the role of the middle section where the threads are intertwined and suspended between the protrusions on either side, emphasizing the consolidation of the threads to fortify the fixing point.

### 1.3 Lateral facial lifting (long length cannula guided U‐type cogged threads)

**FIGURE 2:**
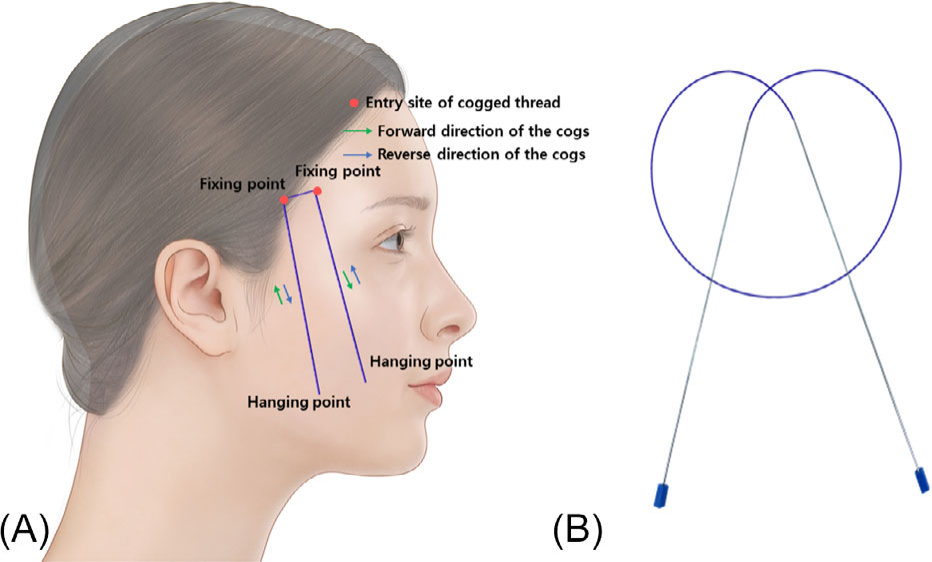
Design of long‐length cannula‐guided U‐type cogged threads (A).The thread depicted in the image is the Secrete line Double S Miracle (B, Hyundai Meditech., Inc., Wonjusi, Republic of Korea).

Similarly, there is a practice among some individuals to refer to a 40 cm or longer cannula‐guided bidirectional cogged thread, often termed as a fixed type thread. When applied in a U‐shaped manner around the zygomatic area, the rationale for directing its central section through the thick deep temporal fascia is to prevent a chiseling effect, wherein the central portion, subject to the weight of the tissues held by the protrusions of both threads, might gradually fray and tear due to the load‐bearing effect. The force applied to seemingly immobilize the thread in place prevents the chiseling effect. This force is the result of the opposing actions of the forces exerted by the protrusions of both threads. The force tending to descend through the forward motion of the left thread's protrusion is counteracted by the reverse force of the right thread's protrusion, and vice versa. Thus, the forward and reverse forces exerted by the protrusions of the left and right threads counteract each other, creating an appearance of the thread being firmly fixed. However, it is essential to note that the central part of the long thread is not truly fixed within the firm tissues of the zygomatic area. Nevertheless, to best withstand the load of the lax tissues held by the lower protrusions, reinforcing the fixing point is essential by ensuring that the upper protrusions closer to the zygomatic area are firmly anchored in the firmer tissues, enabling them to withstand the weight below, ensuring the stability of the upper protrusions without movement (Figure 2).

### 1.4 Lateral facial lifting (long length double needle V‐type cogged threads)

The same principle applies when utilizing V‐shaped or L‐shaped double‐needle long bidirectional cogged threads without a central protrusion. Fundamentally, these threads differ primarily in how they are applied—whether using a cannula to insert the thread or directly inserting it with a needle, along with variations in the length of the central section without protrusions. Consequently, the sensation of the threads not moving within the tissues actually arises from the opposing forces exerted by the forward and reverse directions of the protrusions on the opposite sides, acting in an alternating manner.

**FIGURE 3:**
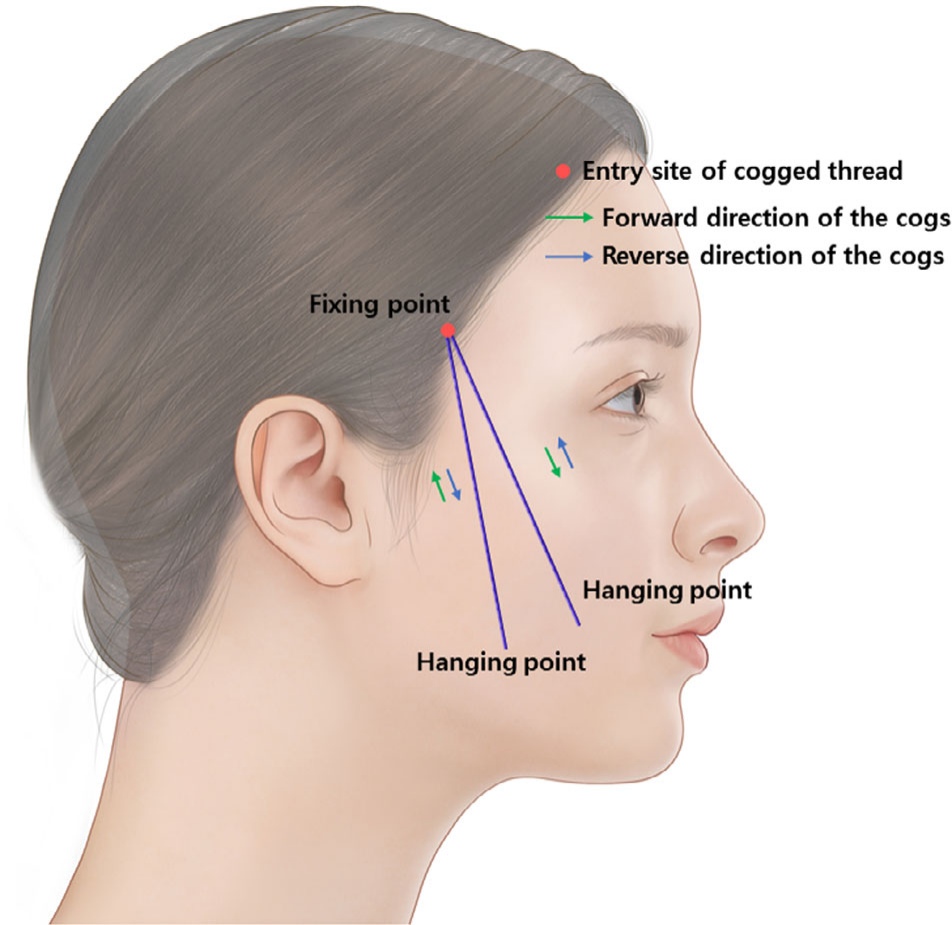
Design of long‐length double needle V‐type cogged threads. The thread depicted in the image is the Secrete line Illusion (Hyundai Meditech., Inc., Wonjusi, Republic of Korea).

However, due to the absence of a lengthy central non‐protruding section, the insertion of these threads typically involves using two entry sites to place the thread through considerably thick tissues, rather than burying it through a single‐entry site. Similarly, ensuring that the protrusions nearer to the entry site are firmly lodged within robust tissue helps counteract the opposing forces generated by the weight of the tissues hanging on the lower protrusions, enhancing the fixing point. This fortification aims to withstand the forces and maintain stability for the upper protrusions without displacement (Figure 3).

### 1.5 Thread lifting for double chin (long length cannula guided or double needle cogged threads)

**FIGURE 4:**
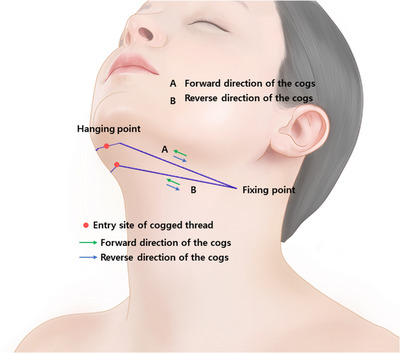
Design for double chin improvement of long‐length cannula guided or double needle cogged threads. The thread depicted in the image is the Secrete Line Double S Miracle and Secrete Line Illusion (Hyundai Meditech., Inc., Wonjusi, Republic of Korea).

In the context of cogged thread procedures, the consistency of tissues engaged by the threads proves to be a more critical factor than the direction of the threads themselves. Even with the same type of cogged thread, variations in the firmness of tissues traversed by the threads influence the location of the fixing point. This mechanism becomes apparent when using elongated bidirectional cogged threads measuring more than 40 cm, particularly when employing a lengthy I‐shaped configuration rather than the U or V shapes in the submental area, as depicted in Figure 4. Upon creating an entry site at the central point, elongated bidirectional cogged threads are introduced bilaterally in an extended I‐shape manner, with both ends maneuvered to traverse the firm tissues below the ears. As previously expounded, the opposing directions of the cogged threads on both sides counterbalance each other, exerting forces primarily on the ends of the thread engaged with the firm tissues instead of the central part, as observed in U or V‐shaped configurations. Consequently, the threads attached to the outer firm tissues act akin to a fixing point, securing the threads and exerting traction on the central section, thereby compressing the loose and irregular tissues hanging in between, thus ameliorating the appearance of the submental area.

### 1.6 Thread lifting for double chin (U‐shape design for double chin improvement of long length cannula guided cogged threads)

**FIGURE 5:**
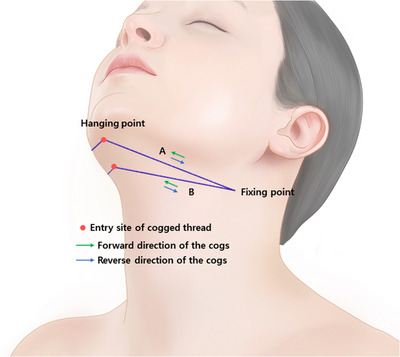
U‐shape design for double chin improvement of long‐length cannula‐guided cogged threads. The thread depicted in the image is the Secrete Line Illusion (Hyundai Meditech., Inc., Wonjusi, Republic of Korea).

The same principle applies when employing elongated U‐shaped bidirectional cogged threads in the submental area and similarly in the central region of the neck using a U‐shaped thread configuration with the absence of a central cog. When inserting threads in such a design, positioning the middle of the thread without cogs facing outward, the ends of the thread, rather than the central part, act as the fixing point. Consequently, the force exerted on the ends of the thread results in an equivalent compression of the irregular central area of the neck, confirming a comparable effect (Figure 5).

## 2 DISCUSSION

The advancement of thread lifting procedures has brought about considerable enhancements in addressing facial sagging, primarily associated with aging. Particularly in the lateral face region, independent of facial expressions, the conventional concepts established in the existing literature are often deemed adequate.^14^, ^15^, ^16^, ^17^ However, the necessity to ensure comfort related to facial expressions has become paramount, warranting procedures that minimize discomfort.^18^ For individuals of Asian descent with prominent cheekbones, accessing the angle between the lateral and anterior faces during procedures posed persistent challenges, prompting the exploration of diverse methods to address these limitations. The recent advancements in thread quality thread lifting companies, coupled with the introduction of varied designs, have significantly contributed to the increased efficacy of thread lifting procedures compared to previous practices.

The primary objective of thread lifting predominantly revolves around addressing skin and connective tissue sagging associated with aging. It has been customary to view facial aging as a universal sagging process affecting all facial structures.

In conclusion, this review delves into the core and general thread lifting procedures, emphasizing the critical role of tissue consistency, anchoring strength, and holding strength.

## CONFLICT OF INTEREST STATEMENT

I acknowledge that I have considered the conflict‐of‐interest statement included in the “Author Guidelines.” I hereby certify that, to the best of my knowledge, that no aspect of my current personal or professional situation might reasonably be expected to significantly affect my views on the subject I am presenting.

## Data Availability

No.
